# Preparation and Physicochemical Properties of the Complex of Naringenin with Hydroxypropyl-*β*-Cyclodextrin 

**DOI:** 10.3390/molecules15064401

**Published:** 2010-06-18

**Authors:** Jiping Wen, Benguo Liu, Erdong Yuan, Yuxiang Ma, Yongyi Zhu

**Affiliations:** 1 College of Grain and Food, Henan University of Technology, Zhengzhou 450052, China; E-Mails: wjp1380@163.com (J.W.); myx366@163.com (Y.M.); zyy4301@163.com (Y.Z.); 2 School of Food Science, Henan Institute of Science and Technology, Xinxiang 453003, China; E-Mail: zzgclbg@126.com (B.L.); 3 College of Light Industry and Food Science, South China University of Technology, Guangzhou 510640, China

**Keywords:** naringenin, hydroxypropyl-β-cyclodextrin, complex, flavonoid

## Abstract

In this study a complex of naringenin with hydroxypropyl-β-cyclodextrin (HP-β-CD) was prepared to improve the hydrophilicity of naringenin. The physicochemical properties of the complex were analyzed by ultraviolet-visible spectrometry (UV), infrared spectrometry (IR), X-ray diffractometry (XRD), differential scanning calorimetry (DSC). The result showed that naringenin had been molecularly dispersed in the HP-β-CD matrix, not forming a new compound and HPLC analysis showed that the solubility of naringenin in water was enhanced from 4.38 μg/mL to 1,272.31 μg/mL.

## 1. Introduction

Flavonoids, rich in fruits, teas, vegetables, and medicinal plants, have received the great attention and been studied extensively, since they have many curative effects such as antibacterial, antioxidant, antiviral, analgesic activities, *etc* [1,2]. However, the poor water and oil solubility of flavonoids limit their application in food and medicine. In our previous works, we proved that the hydrophobicity of flavonoids could be improved by preparing their lecithin complexes [3,4]. 

Naringenin is a flavonoid which is considered to have a bioactive effect on human health as an antioxidant, free radical scavenger, anti-inflammatory, carbohydrate metabolism promoter, and immune system modulator [5]. Since naringenin is insoluble in water at room temperature, in this study we tried to prepare the complex of naringenin with HP-β-CD to improve its water solubility, which could become the basis of the application of flavonoids in food and medicine.

## 2. Results and Discussion

### 2.1. The complex of naringenin and HP-β-CD

**Figure 1:**
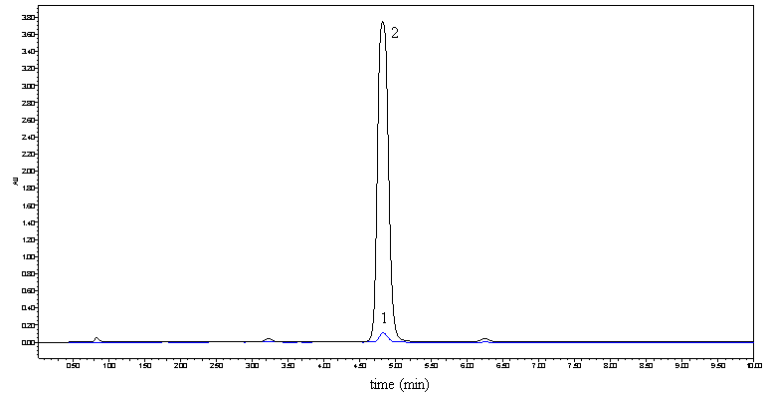
Solubility comparison of naringenin (**1**) and its complex **2** in water.

In previous studies [6,7], Tommasini improved the solubility and dissolution rate of naringenin by complexation with *β*-cyclodextrin and the physicochemical properties of the complex were also investigated by NMR, FT-IR, DSC, X-ray. But due to the low solubility of *β*-cyclodextrin in water (18.5 g/L, 20 °C), the improved solubility of naringenin was still less than 300 μg/mL at 37 °C. Hydroxylpropyl-*β*-cyclodextrin (HP-*β*-CD), a hydroxyalkyl derivative of *β*-cyclodextrin, is an alternative to *α*-, *β*- and *γ*-cyclodextrin (CD), with improved water solubility (>500 g/L, 20 °C). As the first CD derivatives approved by the FDA, HP-*β*-CDs have been widely applied in the food, agriculture and the pharmaceutical fields [8,9]. In this study, we tried to prepare complexes of naringenin with HP-*β*-CD to improve the water solubility of naringenin. The complexes were prepared with different molar ratios of HP-β-CD and naringenin, such as 1, 2, 3, and 4. When the molar ratio was set at 1, all of naringenin could be embedded in HP-β-CD. For the purpose of getting the best quality and use the smallest amount of HP-β-CD, we therefore decided to prepare the complex with a molar ratio of 1. The obtained complex was used for the subsequent analysis. The solubility of the complex in water is shown in Figure 1. By forming the complex, the solubility of naringenin in water increased from 4.38 μg/mL to 1,272.31 μg/mL and the water solubility of naringenin was thus significantly improved.

### 2.2. UV and IR analysis

**Figure 2:**
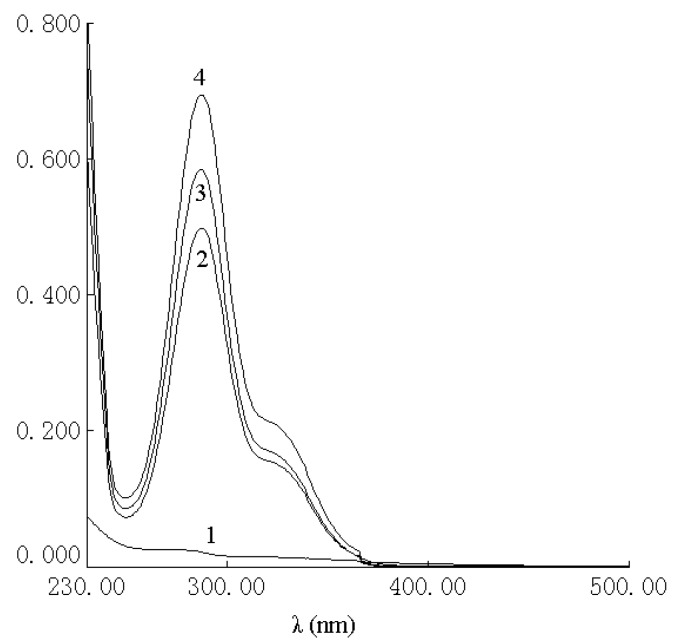
UV spectra of HP-*β*-CD (**1**), complex of naringenin and HP-*β*-CD (**2**), physical mixture of naringenin and HP-*β*-CD (3) and naringenin (4).

**Figure 3:**
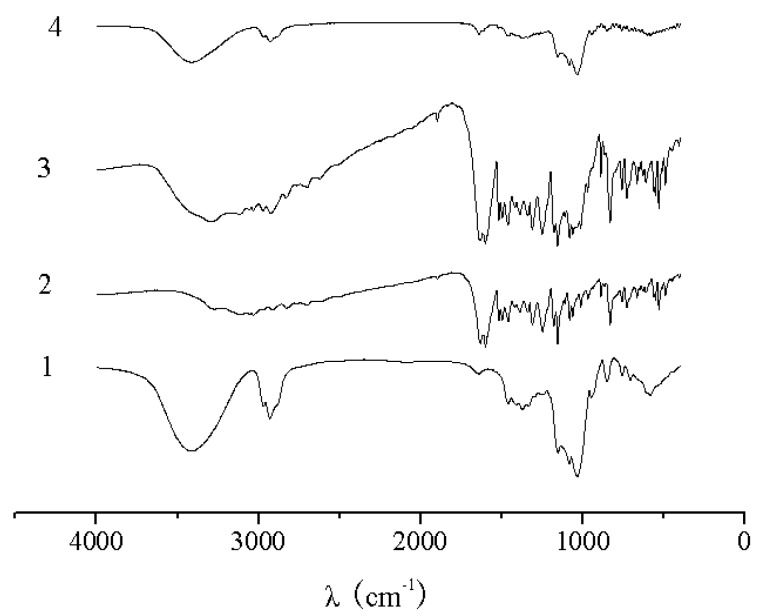
IR spectra of HP-*β*-CD (**1**), naringenin (**2**),physical mixture of naringenin and HP-*β*-CD (**3**) and complex of naringenin and HP-*β*-CD (**4**).

The UV spectra of HP-*β*-CD, naringenin, their physical mixture and the complex are shown in Figure 2. There was no difference between the physical mixture and the complex in the UV analysis. The characteristic absorption peaks of naringenin, the physical mixture and the complex were still present at 287 nm. As shown in Figure 3, the IR spectrum of the physical mixture showed an additive effect of HP-*β*-CD and naringenin. However, in the spectrum of their complex, some small characteristic absorption peaks of naringenin between 500 and 2,000 cm^-1^ were almost masked by those of HP-*β*-CD, and there was no significant difference between the IR spectra of HP-*β*-CD and the complex. From UV and IR spectra, it was concluded that that some weak physical interactions between naringenin and HP-*β*-CD could take place during the formation of the complex.

### 2.3. XRD analysis

**Figure 4:**
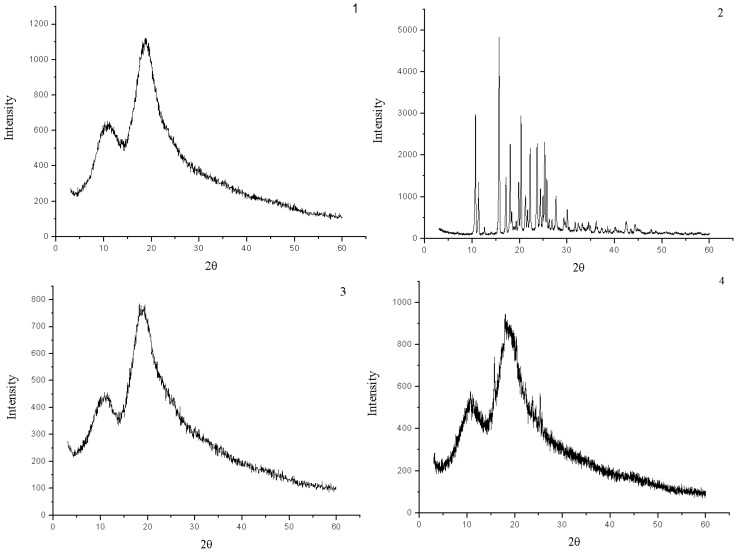
X-ray diffraction patterns of HP-*β*-CD (**1**), naringenin (**2**),complex of naringenin and HP-*β*-CD (**3**) and physical mixture of naringenin and HP-*β*-CD (**4**).

The powder X-ray diffraction patterns of HP-*β*-CD, naringenin, their physical mixture and the complex are shown in Figure 4. The powder diffraction pattern of naringenin displayed sharp crystalline peaks, which is the characteristic of an organic molecule with crystallinity. In contrast, HP-*β*-CD showed an amorphous structure lacking crystalline peaks. Compared with that of the physical mixture, the crystalline peaks had disappeared in the complex. This suggested that naringenin was molecularly dispersed in the HP-*β*-CD matrix, although some crystalline signals of naringenin were still detectable in the physical mixture of naringenin and HP-β-CD.

### 2.4. DSC analysis

**Figure 5:**
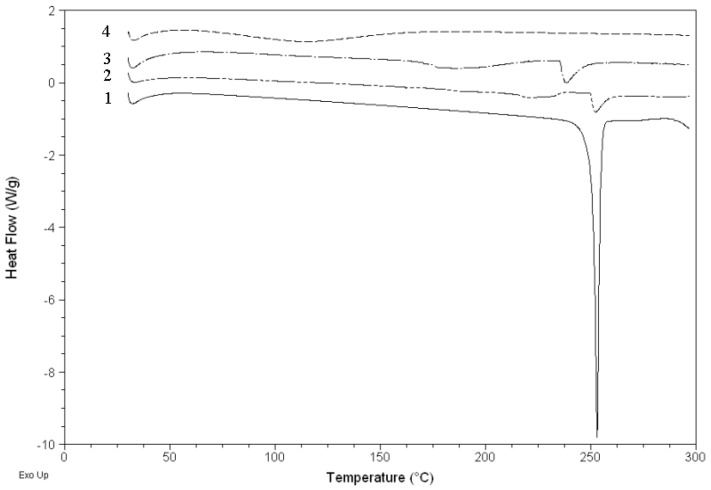
DSC curves of naringenin (**1**), physical mixture of naringenin and HP-*β*-CD (**2**), HP-*β*-CD (**3**) and complex of naringenin and HP-*β*-CD (**4**).

Figure 5 shows the DSC curves of HP-*β*-CD, naringenin, their physical mixture and the complex. The DSC curve of naringenin showed an endothermal peak with an onset temperature at about 251.1 °C, which was attributed to the melting of naringenin. The DSC curve of the physical mixture mainly showed the effects of naringenin and HP-*β*-CD, but the DSC curve of the complex mainly showed the effect of HP-*β*-CD, in which the characteristic endothermal peaks of naringenin disappeared. It was considered that naringenin had been completely dispersed in HP-*β*-CD and they should have some interaction, such as the combination of hydrogen bonds or van der Waals force.

## 3. Experimental

### 3.1. Materials and chemicals

Naringenin (Purity 98%) was purchased from Shaanxi Huike Botanical Development Co., Ltd. HP-*β*-CD (>99%, DS = 5.5) purchased from Wako Pure Chemical Industries, Ltd. (Chuoku, Osaka, Japan). Other chemicals used were of analytical grade.

### 3.2. Preparation of the complex of naringenin with HP-β-CD

Naringenin (0.136 g, 0.5 mM) and HP-*β*-CD (2.8 g, 0.5 mM) were dissolved in ethanol (20 mL) and stirred for 24 h. After the ethanol was removed, the residue was dissolved in water (50 mL) and filtered. The filtrate was frozen at −40 °C for 24 h and then lyophilized (Alpha 1-4, Christ, Germany) and collected. The resultant white power was collected as the complex of naringenin with HP-*β*-CD. 

### 3.3. Preparation of physical mixture of naringenin and HP-β-CD

Naringenin (0.136 g) and HP-*β*-CD (2.8 g) were mixed and stirred in a small beaker at room temperature. The obtained product was collected as the physical mixture of naringenin and HP-*β*-CD.

### 3.4. Solubility in water

Solubility determination of naringenin was carried out by adding excess naringenin and its complex to water (2 mL) at 25 °C. The liquids were agitated for 24 h, and then centrifuged (15 min, 4,000 rpm). The supernatant (500 μL) was diluted to 10 mL with methanol. After ultrasonic treatment, the content of naringenin was determined by HPLC analysis performed on a Waters Alliance HPLC system (USA), which consisted of a Waters 2695 separations module and a Waters 2487 dual λ absorbance detector. The injection volume was 10 μL and the wavelength for detection was set at 288 nm. The samples were separated on a SunFireTM C_18_ reversed phase column (4.6 × 250 mm; 5 μm particle size) made by Waters (USA). The mobile phase consisted of methanol and water (7:3) with a flow rate of 1.0 mL/min. Before HPLC analysis, all samples must be passed through a 0.45 μm Millipore filter. The quantitative analysis of naringenin in the samples was based on an external standard. The chromatographic data were recorded and processed by Empower 2 software.

### 3.5. UV and IR analysis

UV analysis was performed on a TU-1810PC UV spectrophotometer (Purkinje, China) and IR analysis was performed on a TENSOR 27 infrared Spectrophotometer (Bruker, Germany) by the KBr method.

### 3.6. X-ray diffractometry (XRD)

Monochromatic Cu Ka radiation (wavelength = 1.54056 Å) was produced by a D/MAX 2500V/PC X-ray diffractometer (Rigaku Americas Corporation, Japan). The powders of samples were packed tightly in a rectangular aluminum cell. The samples were exposed to the X-ray beam. The scanning regions of the diffraction angle, 2θ, were 5–60˚. Duplicate measurements were made at ambient temperature. Radiation was detected with a proportional detector.

### 3.7. Differential scanning calorimetry (DSC)

The samples sealed in the aluminum crimp cell were heated at the speed of 10 °C/min from 0 to 300 °C in the atmosphere of nitrogen (Q200, TA, USA). The data were recorded and processed by Universal Analysis 2000 software (TA, USA).

## 4. Conclusions

By forming the complex with HP-*β*-CD, the solubility of naringenin in water was significantly improved. According to UV, IR, XRD, DSC analysis, naringenin in the complex was molecularly dispersed in the HP-*β*-CD matrix, and not forming a new compound. The obtained complex could be used in food and medicine.
